# Milk Metabolomics Data Reveal the Energy Balance of Individual Dairy Cows in Early Lactation

**DOI:** 10.1038/s41598-018-34190-4

**Published:** 2018-10-25

**Authors:** Wei Xu, Jacques Vervoort, Edoardo Saccenti, Renny van Hoeij, Bas Kemp, Ariette van Knegsel

**Affiliations:** 1 Adaptation Physiology Group, Department of Animal Sciences, Wageningen University & Research, Wageningen, The Netherlands; 2 Laboratory of Biochemistry, Wageningen University & Research, Wageningen, The Netherlands; 3 Laboratory of Systems and Synthetic Biology, Wageningen University & Research, Wageningen, The Netherlands

## Abstract

In early lactation, dairy cows typically have a negative energy balance which has been related to metabolic disorders, compromised health and fertility, and reduced productive lifespan. Assessment of the energy balance, however, is not easy on the farm. Our aims were to investigate the milk metabolic profiles of dairy cows in early lactation, and to obtain models to estimate energy balance from milk metabolomics data and milk production traits. Milk samples were collected in week 2 and 7 after calving from 31 dairy cows. For each cow, the energy balance was calculated from energy intake, milk production traits and body weight. A total of 52 milk metabolites were detected using LC-QQQ-MS. Data from different lactation weeks was analysed by partial least squares analysis, the top 15 most relevant variables from the metabolomics data related to energy balance were used to develop reduced linear models to estimate energy balance by forward selection regression. Milk fat yield, glycine, choline and carnitine were important variables to estimate energy balance (adjusted *R*^2^: 0.53 to 0.87, depending on the model). The relationship of these milk metabolites with energy balance is proposed to be related to their roles in cell renewal.

## Introduction

In early lactation, the elevated energy requirements for milk production can result in an energy deficit or negative energy balance (NEB) in dairy cows^1,2^. Lipid, glycogen and protein reserves are mobilised to compensate for this energy deficit^3^. A severe NEB is related to a greater risk of metabolic disorders^4^, compromised health and fertility^5^ and a reduced productive lifespan of dairy cows after calving^6^. Therefore, a reliable and early screening of dairy cows with a severe NEB can identify animals with an increased risk for health and fertility problems. Traditionally, the energy balance of dairy cows can be estimated by the difference between energy input (by feed intake) and energy output, based on milk production traits and body weight of the cows^7,8^. On commercial farms, however, feed intake of individual cows is not available. Moreover, calculation of energy balance requires detailed information on net energy derived from feed sources. An alternative way is to estimate energy balance from milk constituents, because a milk sample is easily obtained and milk composition can be daily monitored^9,10^. Recently, extent and duration of negative energy balance has been estimated by measurements of amounts of long-chain fatty acids in milk or δ^13^C in milk fat^11,12^. In previous studies, daily records for milk production traits, including fat yield, protein yield, and fat-to-protein ratio, had a good capability to estimate the energy status at herd level, however, they had limited predictive power (*R*^2^ = 0.40) to estimate the energy balance of individual cows^8,13^.

Metabolomics studies, either untargeted or targeted depending on the type of sample, the type of instrumentation and the approach used, aim to detect and analyse small molecules from bio-fluids. The application of metabolomics techniques and multivariate analysis allows new insights into the metabolic fingerprint of individual animals^14–16^. Milk metabolites are supposed to be derived primarily from the activity of the mammary epithelial cells^17^. Although the biological processes responsible for the milk metabolites is not always completely clear, milk metabolites have been used to study the metabolism of dairy cows or mammary gland function^18–23^. Milk pyruvate concentration and lactate dehydrogenase activity in milk are suggested to be correlated with mammary infections^18,19^, while acetate, butyrate and lactate are related to somatic cell count^20,21^. Milk phosphocholine and glycerophosphocholine have been related to ketosis^22^ and heat stress^23^ in dairy cows. Therefore, milk metabolomic profiles have been identified from dairy cows with different metabolic status. To our knowledge, however, milk metabolomics data have not been used to estimate the energy balance of individual dairy cows.

In this study, we obtained milk metabolomics data, acquired using LC-QQQ-MS, from 31 dairy cows of different parity (*i.e*. number of times a dairy cow has given birth, 2^nd^ or 3^rd^ parity) and with two different dry period lengths (DPL, 0 or 30 days, implying that cows are not milked 0 or 30 days before expected calving date). The approach with a 0-d and 30-d DPL was chosen to create a dataset with a large variation in energy balance among cows^24,25^. Milk samples were collected from these cows in week 2 and 7 after calving to include a week when cows suffer from severe NEB and a week when cows recover from NEB, respectively. Energy balance of individual cows at week 2 and 7 was calculated based on energy intake, body weight and milk energy output. Our first aim was to investigate metabolic profiles in milk of individual dairy cows and to identify the important metabolites that estimate energy balance using partial least squares (PLS) modelling. The second aim was to obtain reduced models to estimate the energy balance of individual cows by a limited number of selected milk metabolites.

## Results and Discussion

### Characterization of Metabolomics Profiles and Multivariate Analysis

In the current study 52 milk metabolites could be reliably identified. The exact origin of milk metabolites is not clear, and they may be secreted from mammary epithelial cells, leaked from damaged somatic cells in the mammary gland or even be transferred from blood^21,26^.

**Figure 1:**
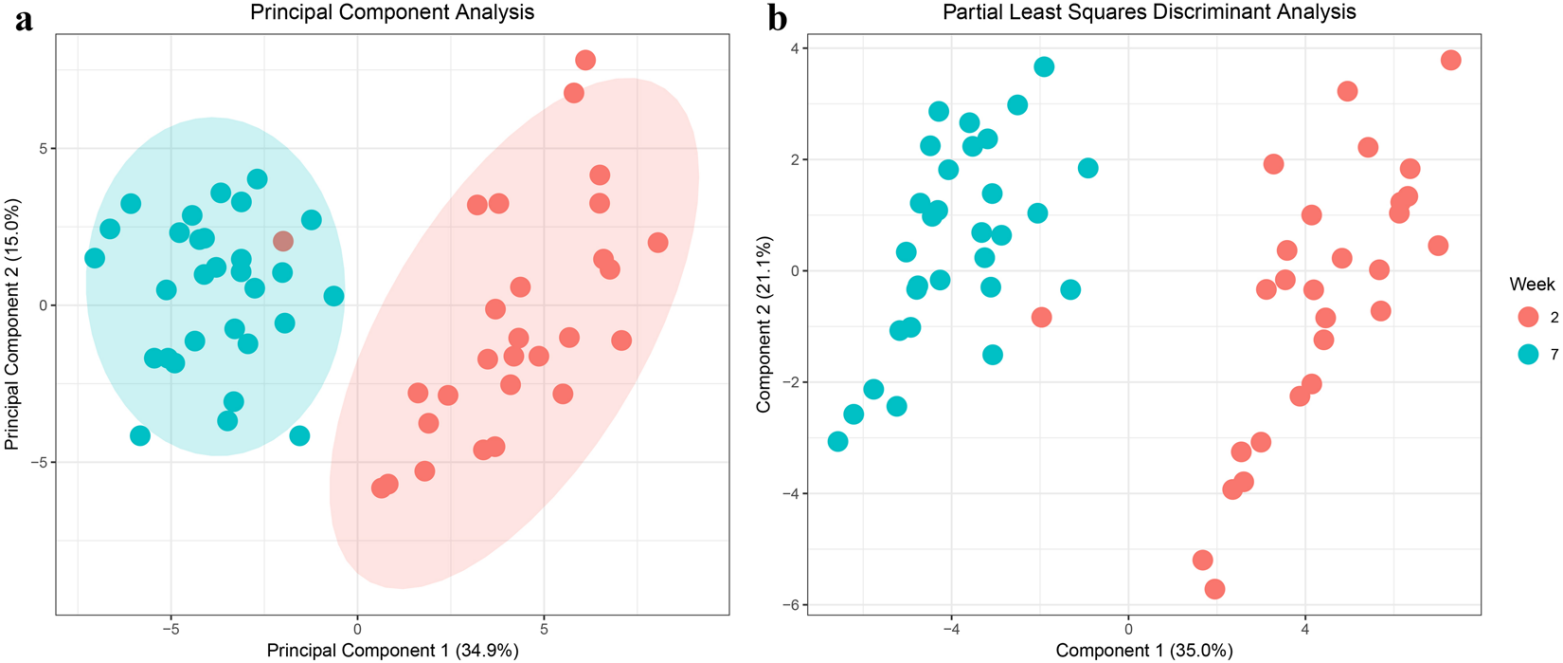
The combined data of milk metabolomics profiles and milk production traits were separated by lactation week in principal component analysis (**a**), and were further discriminated by lactation week with a discriminant power of Q^2^ = 0.86 in partial least squares discriminant analysis (**b**). Numbers in parentheses is the percentage of explained variation of milk metabolites profiles and milk production traits due to separation between week 2 and week 7.

Combined with data from milk production traits, the metabolomics data were subjected to multivariate analysis. Principal component analysis (PCA), on the 52 milk metabolites plus 6 milk production traits showed that the metabolomics profiles can be separated by lactation week but not by dry period length or parity (Fig. 1a and Supplementary Fig. S1). Partial least squares discriminant analysis (PLS-DA) confirmed that data could be discriminated by lactation week (Fig. 1b) with a discriminant power of Q^2^ = 0.85, (*P*-value = 2.0 × 10^−4^ obtained using a permutation test). A value of Q^2^ = 1 indicates a perfect discrimination between lactation week 2 and 7.

**Figure 2:**
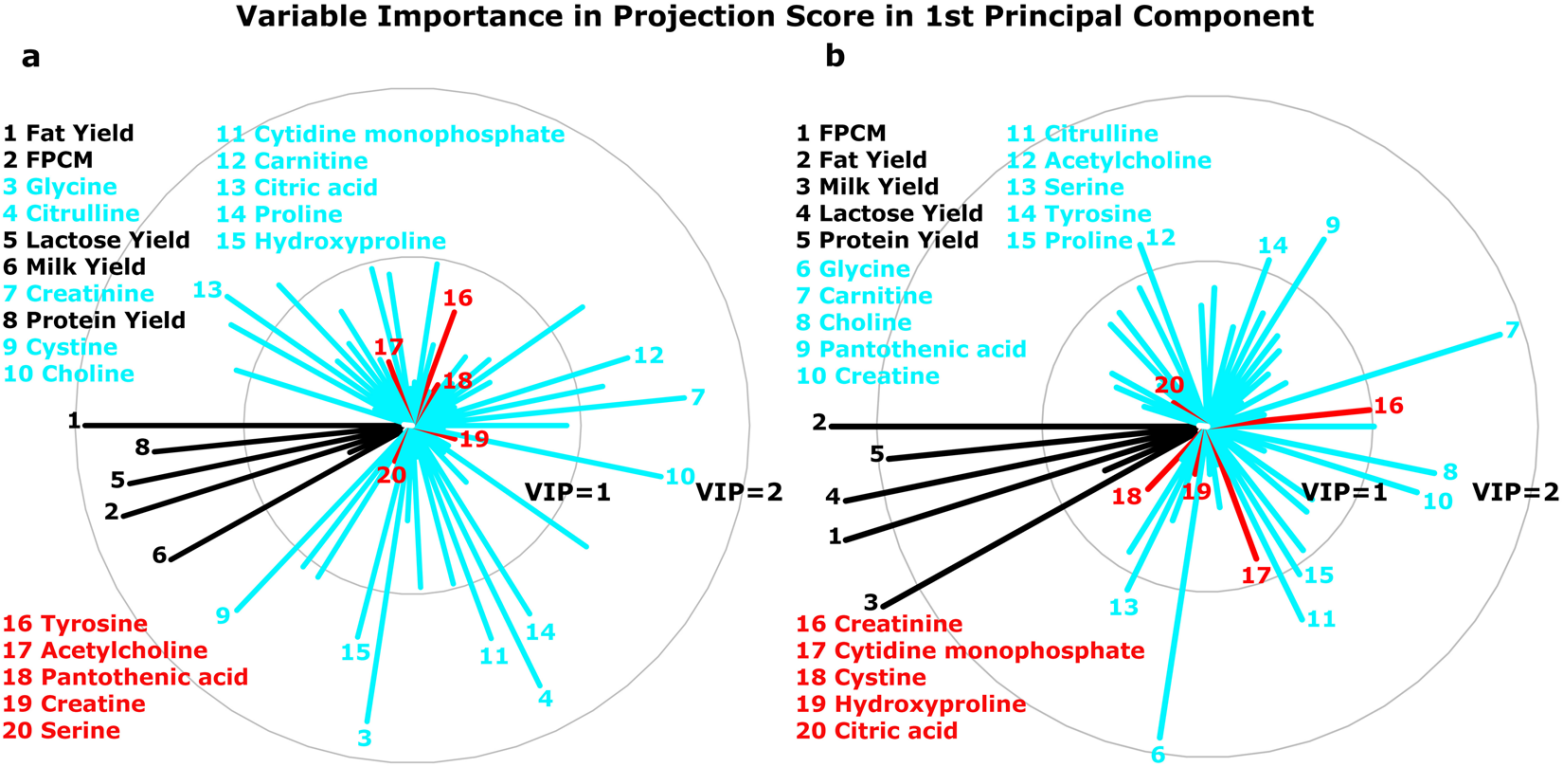
Variable importance in projection (VIP) scores in the first principal component calculated by partial least squares (PLS) to estimate the energy balance of dairy cows in lactation week 2 (**a**) and week 7 (**b**). The top 15 metabolites with relatively higher VIP score are shown. The relative higher VIP score means that variable has higher capability to predict energy balance in PLS analysis. Black line and text represent milk production traits, blue line and text represent milk metabolites with relatively higher VIP score, and red line and text represent milk metabolites which were in the top 15 in one week, but not in the other week. Abbreviation: FPCM, fat- and protein-corrected milk.

Given the data could be separated by lactation week, further analysis was performed considering data for lactation week 2 and 7 separately. Using PLS regression, energy balance was used as the dependent variable Y, and milk yield, fat yield, protein yield, lactose yield, urea, fat- and protein-corrected milk (FPCM)^27^ and 52 milk metabolites were used as predictor variables X. The capability of the PLS models to estimate the energy balance were Q^2^ = 0.72 and Q^2^ = 0.84, for the PLS models for week 2 and 7 respectively, and both models were statistically significant (*P*-value = 2.0 × 10^−4^ obtained using a permutation test). The variable importance in projection (VIP) scores of all variables in the first component of PLS for week 2 and 7 are shown in Fig. 2. Five milk production traits (milk yield, fat yield, protein yield, lactose yield and FPCM) and five milk metabolites (glycine, choline, carnitine, citrulline and proline) were selected from top 15 variables with the highest VIP scores in both lactation weeks.

### Correlation analysis

**Figure 3:**
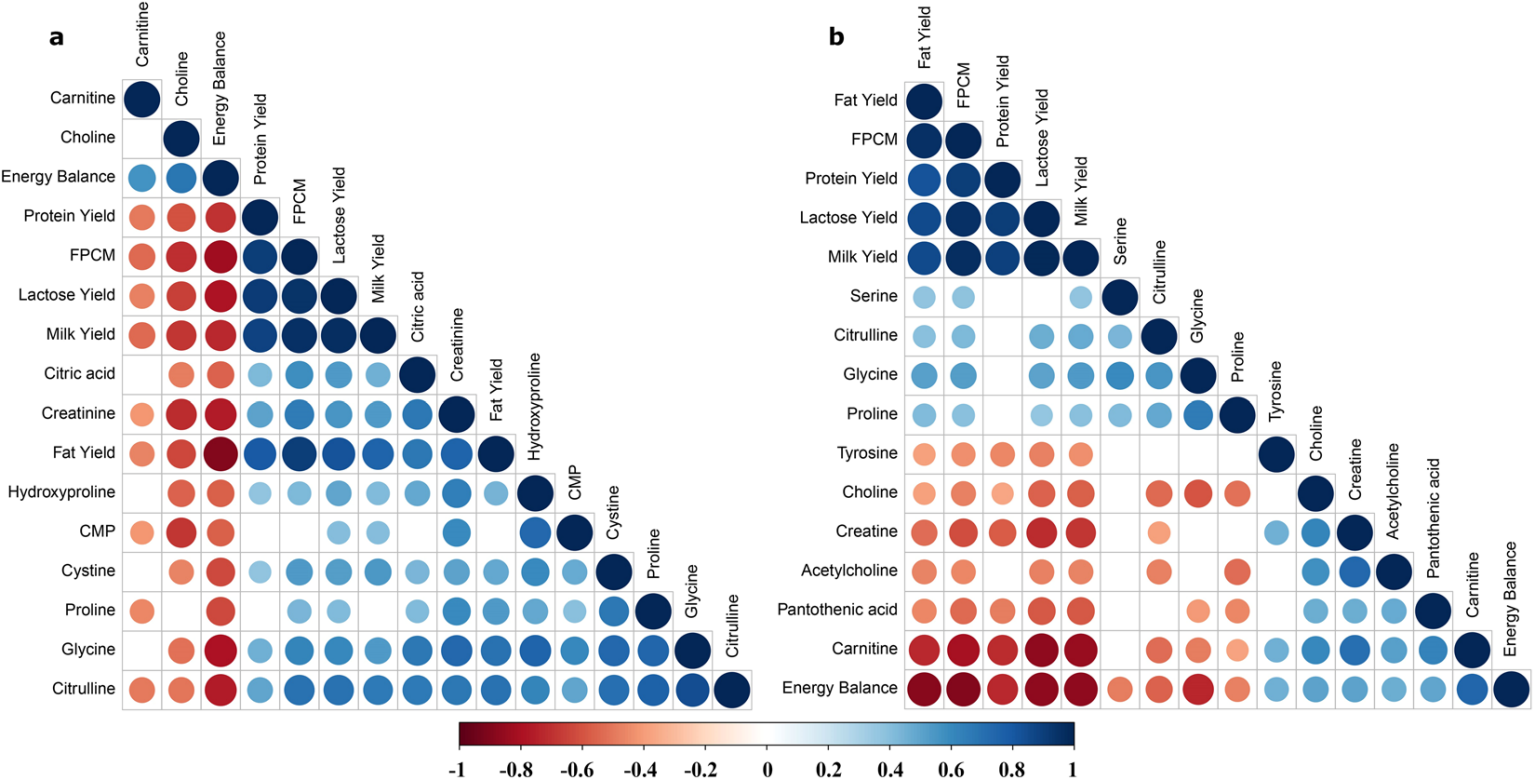
Pearson correlations matrix between 15 variables in milk with the relatively higher variable importance in projection score from partial least squares analysis and energy balance of dairy cows in lactation week 2 (**a**) and week 7 (**b**). The size of dots is proportional to the absolute value of correlations, the blue and red colour represent positive and negative direction of correlations, respectively, and blank represents that there was no correlation between two variables (*P*-value > 0.05). Abbreviations: CMP, cytidine monophosphate; FPCM, fat- and protein-corrected milk.

In the current study, the energy balance of dairy cows was calculated, and correlations between energy balance and milk metabolites were analysed. Figure 3 shows Pearson correlations among the top 15 variables with relatively higher VIP score for the first principal component in the PLS model to estimate energy balance of dairy cows in lactation week 2 and 7. The complete correlation matrix of 52 milk metabolites and 6 milk production traits with energy balance is shown for lactation week 2 and 7 separately in Supplementary Fig. S2. All top 15 variables in the PLS model were correlated with energy balance in lactation week 2, the correlation coefficient (r) ranged from −0.88 to −0.55, or 0.56 to 0.71 (Fig. 3a). The same trend was observed in lactation week 7 (Fig. 3b). In earlier studies, milk metabolites were identified as indicators for milk characteristics and metabolic status of dairy cows. Choline, carnitine, citric acid and lactose in milk were correlated with coagulation properties of milk^28^. Increased beta-hydroxybutyrate concentration in milk was related to subclinical ketosis of dairy cows^29^. In addition, an observed correlation between an amino acid, lysine, and protein content in milk was proposed to relate to the limiting nature of amino acids for protein production^30^. In our study, five milk production traits, milk yield, fat yield, protein yield, lactose yield and FPCM, were clearly correlated (*P*-value < 5.0 × 10^−2^) with each other in both lactation weeks. The correlation between top 15 variables in milk and energy balance suggests a relationship of biological pathways involved in energy metabolism and cell regeneration, which will be discussed in more detail below.

### The effect of dry period length, parity and energy balance on milk metabolites and milk production traits

The effect of DPL, parity and energy balance on milk metabolites and milk production traits of dairy cows in lactation week 2 and 7 was analysed with a mixed model. Briefly, DPL, parity, energy balance and their two-way interactions were included in the model. Significant two-way interactions (*P*-value < 5.0 × 10^−2^) were kept in the model (Supplementary Figs S3 and S4).

Dairy cows with a 0-d dry period had a lower lactose and protein yield than cows with a 30-d dry period in week 2 after calving (Table 1), which is in line with earlier studies^25,31^. The lower lactose and protein yield was probably due to the lower milk yield of cows with a 0-d dry period, compared with cows with a 30-d dry period^25^. The effect of DPL on milk yield depended on the energy balance (*P*-value = 3.1 × 10^−3^) of cows, cows with a 30-d had a higher milk yield than cows with a 0-d dry period only when the energy balance was more than −125.0 kJ/kg^0.75^ per day. Also, protein yield, lactose yield and FPCM were affected by an interaction between DPL with energy balance of cows (Supplementary Fig. S3). In week 7, dairy cows with a 0-d dry period had a higher concentration of acetylcholine, pantothenic acid and tyrosine than cows with a 30-d dry period (Table 2). The two-way interaction between DPL and energy balance affected citrulline, glycine, protein yield and FPCM. The effect of DPL had the same direction to milk metabolites or milk production traits on different energy balance states in lactation week 2 and week 7 (Supplementary Figs S3 and S4). In earlier studies, the lower milk yield after shortening (28 to 30-d) or omitting (0-d) dry period improved energy balance and metabolic status in dairy cows in early lactation, compared with a conventional dry period of (56 to 60-d)^24,25^. Because in earlier studies specifically the contrast in energy balance between 0-d and 30-d DPL was large, compared with a DPL of 60-d^24,25^, the current study used data and samples of cows with either a 0-d or 30-d DPL. Also in the current study, cows with a 0-d dry period had a less severe negative energy balance than cows with a 30-d dry period (−77.2 vs. −276.8 kJ/kg^0.75^ per day for 0-d vs. 30-d dry period in week 2, *P*-value = 6.7 × 10^−3^; and 112.7 vs. −27.3 kJ/kg^0.75^ per day for 0-d vs. 30-d dry period in week 7, *P*-value = 2.5 × 10^−2^). Moreover, a 0-d DPL not only affects the energy balance in early lactation, but also affects mammary cell regeneration^32^ and mammary health^33^, which might contribute to differences in milk metabolic profiles among cows with different dry period lengths. To limit this possible effect of DPL on milk metabolites, the relation between EB and individual milk metabolites was corrected for DPL in Tables 1 and 2.

**Table 1:** The effect of dry period length (DPL), parity and energy balance (EB), on 10 milk metabolites and 5 milk production traits obtained through partial least squares analysis in lactation week 2. These 15 variables had a relatively higher VIP score^1^ in week 2.

	DPL		Parity		EB		Two-way interaction	
	FC^a^	*P*-value	FC^b^	*P*-value	*P*-value	EB*DPL	EB*Parity	DPL*Parity
**Milk metabolites**								
Carnitine	1.19	0.80	1.28	0.52	<0.01	NM^2^	NM	NM
Choline	1.41	0.35	1.51	<0.01	<0.01	NM	0.05	NM
Citric acid	0.80	0.12	0.93	0.94	<0.01	NM	NM	0.08
Citrulline	0.69	0.92	0.90	0.12	<0.01	NM	NM	NM
Creatinine	0.86	0.94	0.85	0.43	<0.01	NM	NM	0.02
Cystine	0.70	0.68	0.78	0.84	<0.01	NM	NM	NM
CMP^3^	0.62	0.77	0.74	0.14	0.02	NM	0.08	NM
Glycine	0.54	0.33	0.71	0.13	<0.01	0.08	<0.01	0.10
Hydroxyproline	0.65	0.73	0.90	0.54	<0.01	NM	0.10	NM
Proline	0.94	0.65	0.98	0.88	<0.01	NM	NM	NM
**Milk Production Traits**								
Fat (kg/d)	0.81	0.39	0.85	0.20	<0.01	0.07	NM	0.01
FPCM^4^	0.85	0.07	0.83	0.55	<0.01	<0.01	NM	0.08
Lactose (kg/d)	0.87	0.05	0.87	0.14	<0.01	<0.01	NM	NM
Milk Yield (kg/d)	0.88	0.13	0.82	0.84	<0.01	<0.01	NM	NM
Protein (kg/d)	0.86	<0.01	0.89	0.12	<0.01	<0.01	NM	0.06

^a^Fold change in the metabolite concentration (DPL 0/30).

^b^Fold change in the metabolite concentration (parity 2/3).

^1^Variable importance in projection score in partial least squares analysis.

^2^NM: Not included in model.

^3^CMP: Cytidine monophosphate.

^4^FPCM, fat- and protein-corrected milk.

**Table 2:** The effect of dry period length (DPL), parity and energy balance (EB), on 10 milk metabolites and 5 milk production traits obtained through partial least squares analysis in lactation week 7. These 15 variables had a relatively high VIP score^1^ in week 7.

	DPL		Parity		EB		Two-way interaction	
	FC^a^	*P* value	FC^b^	*P* value	*P* value	EB*DPL	EB*Parity	DPL*Parity
**Milk metabolites**								
Acetylcholine	1.73	<0.01	1.07	0.94	0.15	NM^2^	NM	NM
Carnitine	1.21	0.19	1.17	0.03	<0.01	NM	NM	NM
Choline	1.23	0.15	1.27	0.01	<0.01	NM	NM	NM
Citrulline	0.83	0.21	0.97	0.99	<0.01	<0.01	NM	0.03
Creatine	1.17	0.05	1.13	0.05	0.05	NM	NM	NM
Glycine	0.70	0.12	0.76	0.02	<0.01	<0.01	<0.01	NM
Pantothenic acid	1.32	<0.01	1.18	0.04	0.10	NM	NM	NM
Proline	0.88	0.35	0.85	0.22	0.02	NM	NM	NM
Serine	0.87	0.93	0.87	0.36	0.02	NM	NM	NM
Tyrosine	1.03	0.03	1.05	0.13	<0.01	0.02	<0.01	<0.01
**Milk Production Traits**								
Fat (kg/d)	0.84	0.52	0.92	0.47	<0.01	NM	NM	NM
FPCM^3^	0.86	0.69	0.88	0.15	<0.01	0.02	NM	NM
Lactose (kg/d)	0.82	0.86	0.85	0.03	<0.01	0.09	NM	NM
Milk Yield (kg/d)	0.84	0.95	0.83	<0.01	<0.01	NM	NM	NM
Protein (kg/d)	0.91	0.25	0.89	0.08	<0.01	<0.01	NM	NM

^a^Fold change in the metabolite concentration (DPL 0/30).

^b^Fold change in the metabolite concentration (parity 2/3).

^1^Variable importance in projection score in partial least squares analysis.

^2^NM: Not included in model.

^3^FPCM, fat- and protein-corrected milk production.

Parity had an effect on milk metabolites and milk production traits of dairy cows in both lactation weeks. In week 2, young cows (parity 2) had a higher choline concentration than older cows (parity 3). In week 7, young cows had higher choline, carnitine and pantothenic acid concentration, but lower glycine concentration, milk yield and lactose yield, compared with older cows. Therefore, parity affected more milk metabolites of dairy cows in week 7 than in week 2. Young cows had a different energy balance than older cows in week 2 (−93.8 *vs*. −249.1 kJ/kg^0.75^ per day for young *vs*. older cows, *P*-value = 3.3 × 10^−2^). Both young and older cows recovered from NEB in week 7 (67.9 *vs*. 17.5 kJ/kg^0.75^ per day for young *vs*. older cows, *P*-value = 4.1 × 10^−1^). The different energy balance of young cows in week 2 is explained by the lower milk production (FPCM), compared with older cows (29.6 *vs*. 35.9 kg/d for young *vs*. older cows, *P*-value = 1.7 × 10^−2^) at a similar net energy intake for lactation (120.15 *vs*. 118.04 MJ/d for young *vs*. older cows, *P*-value = 0.67).

In each one of lactation week 2 and 7, 10 milk metabolites and 5 milk production traits with relatively higher VIP score were related to the energy balance in the PLS models. It could be expected that the severity in NEB is related to the metabolic profile in milk of dairy cows, although little information has been available hitherto on this relationship. In an earlier study, we proposed that the presence of sugar phosphates in milk of cows during severe NEB indicated leakage of these components from mammary epithelial cells into milk due to apoptosis^34^.

### Estimating the energy balance using milk metabolites and milk production traits

The aim of this study was to estimate the energy balance of dairy cows by reduced models using the milk metabolites and milk production traits. Models 1-6 were obtained through top 15 variables with relatively higher VIP score in lactation week 2 or week 7, and models 7–8 were obtained through all variables with relatively higher VIP score for both week 2 and week 7 (Table 3). The reduced models were selected based on: (*i*) the highest adjusted *R*^2^; and (*ii)* number of independent variables in the model is determined by “one in ten rule”, in other words, for each ten observations, one variable is allowed in the model^35^. Model 1, 4 and 7 included only milk metabolites to estimate the energy balance in week 2 or week 7, or combined data of week 2 and 7, respectively. Model 2, 5 and 8 aimed to estimate the energy balance with both milk metabolites and milk production traits included in week 2 or week 7, or combined data of week 2 and 7, respectively. Model 3, 6 and 9 included only milk production traits to estimate the energy balance in week 2 or week 7, or combined data of week 2 and 7, respectively.

**Table 3:** Reduced models to estimate the energy balance of dairy cows in lactation week 2 and 7. The reduced models were selected by multivariate linear regression.

Model composition	Model no.	Model (Equation)	*R* ^2 1^	adjusted *R*^2 2^
**Dataset of dairy cows in week 2**				
M^3^	**1**	EB = −357.2–1.9*Glycine (60.0%) + 0.5*Choline (21.6%) + 1.6*Carnitine (18.4%)	0.72	0.68
M + P^4^	**2**	EB = 222.0–288.2*Fat (65.8%) + 0.3*Choline (18.0%) − 1.2*Glycine (16.1%)	0.85	0.83
P^5^	**3**	EB = 580.7–532.4*Fat	0.79	0.78
**Dataset of dairy cows in week 7**				
M	**4**	EB = −204.3–3.2*Glycine (60.7%) + 1.9*Carnitine (29.9%) + 35.2*Tyrosine (9.4%)	0.69	0.65
M + P	**5**	EB = 591.3–334.2*Fat (63.9%) − 2.4*Glycine (30.3%) + 28.7*Tyrosine (5.9%)	0.89	0.88
P	**6**	EB = 632.2–331.4*Fat (46.4%) − 14.9*Milk Yield (37.8%) + 338.9*Protein (15.8%)	0.81	0.80
**Dataset of dairy cows in both week 2 and 7**				
M	**7**	EB = −178.5–2.6*Glycine (56.8%) + 2.1*Carnitine (43.2%)	0.77	0.76
M + P	**8**	EB = 222.9–301.3*Fat (48.8%) − 1.7*Glycine (34.1%) + 1.1*Carnitine (8.6%) + 5.2*Citric acid (8.5%)	0.88	0.87
P	**9**	EB = 613.4–648.0*Fat (82.6%) + 653.4*Lactose (11.4%)–21.6*Milk Yield (6.0%)	0.55	0.53

The models were built by the concentrations of the milk metabolites and milk production treats. ^1^*R*^2^ was obtained through 10-fold cross-validation.

^2^adjusted *R*^2^ considered the number of independent regressors in a model, and it was obtained through formula, adjusted *R*^2^ = 1 − [(1 − *R*^*2*^) (n − 1)/(n − k − 1)], n is the number of sample size, k is the number of independent regressors, excluding the constant.

^3^M: only milk metabolites are used in the model.

^4^M + P: both milk metabolites and milk production traits are used in the model.

^5^P: only milk production traits are used in the mode.

The models (2, 5 and 8) using both milk metabolites and milk production traits had the highest capacity to estimate the energy balance of dairy cows. In both week 2 and 7, our models based on milk metabolites had slightly lower estimating capacity than our models based on milk production traits (adjusted *R*^2^ = 0.68 *vs*. 0.78 in week 2; adjusted *R*^2^ = 0.65 *vs*. 0.80 in week 7). In earlier work, milk production traits, including, milk yield, fat, protein and lactose yield, were related to the energy output of dairy cows^36,37^. In week 2, dairy cows were suffering from severe NEB, energy balance = −177.0 kJ*/*kg^0.75^ per day, while cows were recovering from NEB in week 7, energy balance = 42.7 kJ/kg^0.75^ per day (*P*-value = 1.5 × 10^−5^). The difference in energy balance between lactation weeks indicate that dairy cows were also in different metabolic status in lactation week 2, compared with week 7. A higher amount of body reserves can be hypothesised to be mobilised for milk synthesis in week 2 than in week 7, which may explain why the capacity of milk production traits to estimate the energy balance only in week 2 is greater than when both weeks are combined. In earlier studies, milk production traits were used as promising indicators for energy balance at herd level and subclinical ketosis of individual cows^38,39^. Milk fat-to-protein ratio increased coefficients by 19.0 to 52.0% when it was used to estimate herd-level energy balance at herd level^40^. Also in this study, fat yield contributed significantly to the estimation of the energy balance of individual cows (Table 3). Fat yield accounted for 65.8% and 63.9% of the variation of energy balance in models 2 and 5, respectively. Increasing fat yield resulted in greater energy output of dairy cows in early lactation^1,8^, which accounted for the lower energy balance and the role of fat yield in reduced models to estimate the energy balance where milk production traits were involved. Fat yield in model 8 accounted for 48.8% explained variation for energy balance. Glycine, carnitine and citric acid together accounted for more explained variation to estimate energy balance, 34.1, 8.6 and 8.5%, respectively, than fat yield alone. Compared with models 2 and 5, the variation explained by fat yield decreased in model 8. The lower variation by fat yield in model 8 was in line with the low coefficient to estimate energy balance in model 9 by milk production traits only. Glycine was an important metabolite to estimate the energy balance in all models with milk metabolites, next to carnitine, choline, tyrosine and citric acid. The possible biological relationship between glycine and the energy balance in early lactation is discussed in more detail below. Moreover, reliable and practical tests should be developed to detect these milk metabolites to estimate the energy balance under practical conditions.

### Energy balance and biological pathways

In early lactation, dairy cows mobilize their body reserves to meet the energy requirement for milk synthesis and secretion in the mammary gland^41,42^. Therefore, the energy balance of individual cows could be expected to be reflected in metabolic patterns in milk. In previous studies, metabolic changes were observed in early lactation of cows as compared with late lactation^43,44^. Klein and co-workers observed that 19 amino acids, glucose and a number of carboxylic acids were related to ketosis in dairy cows^37^. Ketosis indicates the NEB in cows, but its relationship with milk metabolites is not fully clear. Klein *et al*. observed a positive correlation of plasma glycine-to-alanine ratio to milk ketone bodies, acetone (*r* = 0.77) and BHBA (*r* = 0.64), suggesting an excessive protein mobilization and a shortage in glucose supply. Klein *et al*. proposed that a shortage of vitamin B6 in these animals could be the reasoning for these biological effects^43^. Meijer *et al*. observed large changes in amino acids in muscle and plasma of high yielding dairy cows in early lactation, suggesting that protein was degraded for the supply of amino acids to the udder^38^. It was proposed that glutamine is potentially limiting for protein synthesis. Although these and similar studies have been done carefully, they do not provide a clear biological relationship of energy balance with metabolism of individual cows. In our study, we calculated energy balance and measured metabolic variables in milk of individual cows.

**Figure 4:**
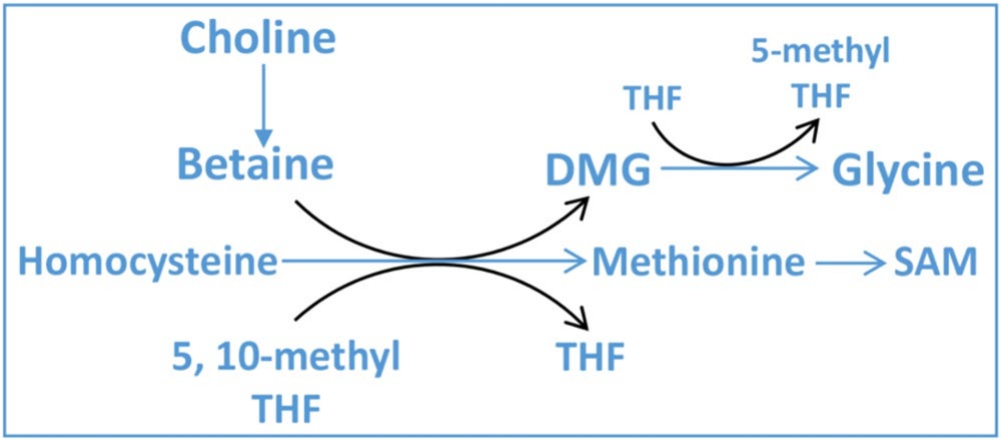
As a major methyl donor from diet, choline transfers its methyl-group to SAM (S-adenosyl methionine) via betaine with a concomitant formation of glycine in this process. The figure was adapted from Friesen *et al*.^46^. Abbreviations: THF, tetrahydrofolate; DMG, dimethylglycine.

**Figure 5:**
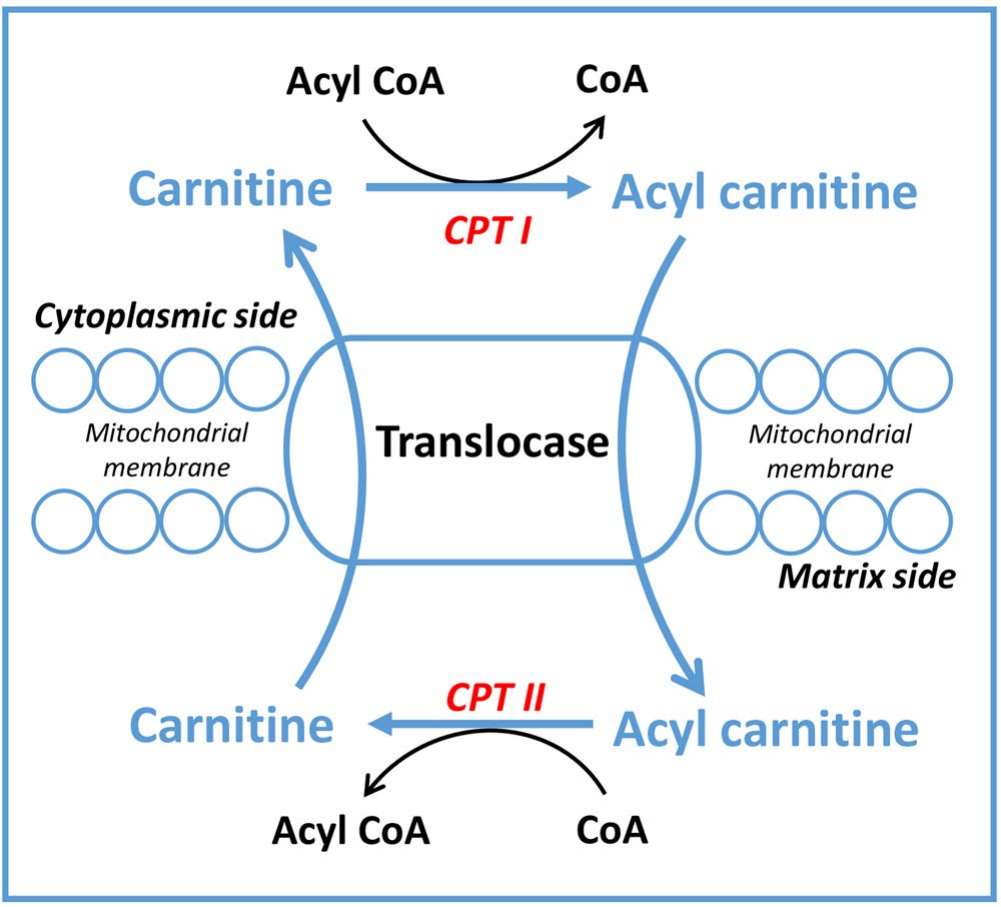
Fatty acid transportation mechanism in the cell. The inner mitochondrial membrane is impermeable to fatty acids and a specialized carnitine carrier system operates to transport activated fatty acids from cytosol to mitochondria. Carnitine is converted to acyl carnitine for fatty acid transportation. The figure was adapted from Nelson *et al*.^66^. Abbreviations: CoA, coenzyme A; CPT I, Carnitine palmitoyltransferase I; CPT II, Carnitine palmitoyltransferase II.

Of the milk metabolites observed in our study, a large number of metabolites were correlated, either positively or negatively, to the energy balance of the individual cows (Fig. 2). Some of these metabolites could be involved in the same metabolic pathway which can be extrapolated from the strong correlations between some components as observed in Fig. 3. We therefore created reduced models in order to include metabolites with a limited interdependence (Table 3). Of the metabolites in our reduced models, glycine, choline, carnitine, citric acid and tyrosine were the ones which were most clearly related to the energy balance. Glycine was found to be the most important metabolite in all models. In previous studies, both plasma and milk glycine concentrations were increased during early lactation of cows, compared with late lactation^43,44^. In the current study, milk glycine was negatively related to energy balance in both lactation week 2 (*r* = −0.80) and week 7 (*r* = −0.74) (Fig. 3). Besides glycine also choline is related to energy balance (Table 3). These two metabolites, glycine and choline, are both important in the one carbon metabolism (Fig. 4). In the one carbon metabolism dietary choline is a methyl donor for important biological processes involving the folate cycle, redox balance status and cell renewal^45,46^. Choline is regarded as a limiting nutrient for transition dairy cows^47,48^. In earlier studies, dairy cows supplied with rumen-protected choline had increased milk production^49^ with a decreased incidence of diseases, including fatty liver^50^, ketosis and mastitis^51^. These earlier studies tested the effect of choline on animal health and milk production, however, no relationship to energy balance was shown. In our study we observed a choline deficiency in severe NEB cows which could explain the low production and high disease incidence in early lactation cows. In our study, choline is not only negatively correlated with glycine, but also positively correlated with carnitine (Fig. 3). The question is how these three small metabolites, glycine, choline and carnitine are mechanistically related to energy balance. Cows with severe negative energy balance have high concentrations of glycine in milk, and these cows have low levels of choline and carnitine in milk (Supplementary Fig. 3). In cows with mild energy balance the opposite is observed for these three metabolites, low levels of glycine and high levels of choline and carnitine. Carnitine is a quaternary ammonium salt as is choline. Carnitine is involved in fatty acid metabolism (Fig. 5) and is not directly involved in one carbon metabolism. However, as carnitine is synthesized in the liver from lysine and methionine a possibly relationship to one carbon metabolism could exist. On the other hand, it has been proposed that a major source of carnitine originates from protein lysine N-trimethylation on release from proteins by protein hydrolysis^52^. The low levels of carnitine in negative energy balance cows could therefore be the result of low carnitine synthesis in the liver or from low levels of protein hydrolysis. However, low levels of carnitine could be also the result of high levels of acylcarnitines. Formed in lipid and amino acid oxidation processes, acylcarnitines are intermediates in the breakdown processes of lipids and amino acids. Giesbertz *et al*. quantitatively measured the concentration of 56 acylcarnitines in mice with a metabolic syndrome^53^. Unfortunately, we did not measure acylcarnitines in our study. However, no relationship between acetylcarnitine (which we could measure in our LC-QQQ-MS setup), carnitine or energy balance was found. It can be expected that more lipid metabolism occurs in cows with NEB as these cows have higher fat yield in milk, with a concomitant increase of acylcarnitines. Future studies have to show if the low levels of carnitine in negative energy balance cows are related to low levels of biosynthesis of carnitine or high levels of acyl-carnitines. From our findings that in severe negative energy balance glycine levels are relatively high in concentration and methylated metabolites as choline, and carnitine are low in concentration, we conclude that severe NEB cows have a need for methyl donor compounds. This observation mimics observations made in cancer cells, where there is a high demand for methyl donors^54,55^. Labuschagne *et al*. observed that in cancer cells there is a high demand for methyl donors and that these methyl groups are used in nucleic acid synthesis^54^. Interestingly, glucose was observed in our study to be related to energy balance (low levels of glucose in NEB cows). We propose that glucose is used additionally as methyl donor via the glucose-serine pathway, and not only for lactose production^56^. This reflects observations made in oncology where fast dividing cells use glucose in what is called aerobic glycolysis (the Warburg effect). Glucose was originally proposed by us to be important for the C2/C3 compound ratio in early lactation of cows but possibly the low levels of glucose in NEB cows are also related to the one-carbon metabolism^56,57^.

Finally, we also observed relationships between amino acids and negative energy balance. These amino acids are highly correlated and in our reduced model tyrosine was selected. In the current study, cows with a NEB had low levels of tyrosine in milk. As the energy balance was negatively correlated with protein yield in both lactation week 2 and 7, we propose that the lower levels of amino acids in milk are related to higher levels of protein synthesis.

## Conclusions

In the current study, 52 milk metabolites of cows during a status of negative energy balance were detected through LC-QQQ-MS. The energy balance had a high correlation with specific milk metabolites and milk production traits in both lactation week 2 and week 7. Nine reduced models were composed to estimate the energy balance of dairy cows in lactation week 2 and 7, with a range from 53% to 88% predicting power. Both milk metabolites and milk production traits had an important role in these models, in particular glycine, choline, carnitine and fat yield. The strong relationship of these metabolites with energy balance of dairy cows in early lactation could be related with their roles in cell renewal.

## Material and Methods

### Animals and experimental design

The experimental protocol for the study was approved by the Institutional Animal Care and Use Committee of Wageningen University and was conducted at Dairy Campus research farm (WUR Livestock Research, Lelystad, the Netherlands) in accordance with the relevant guidelines and regulations. The experimental design was described previously^58^. Briefly, 31 high-yielding Holstein-Friesian dairy cows averaging 637.4 ± 67.1 kg of body weight (in week 2 after calving) participated in this study. Cows were selected from two parities (2nd or 3rd parity) and randomly assigned to one of two DPL (0 or 30 days) before calving. Prepartum, cows with a 0 day DPL received a lactation ration based on grass silage and corn silage (6.4 MJ net energy for lactation (NE)/kg dry matter (DM)). Cows with a 30 days DPL received a dry cow ration based on grass silage, corn silage and wheat straw (5.4 MJ NE/kg DM). Postpartum, all cows received the same basal lactation ration as provided to lactating cows with a 0 day DPL prepartum plus additional concentrates. Postpartum, concentrate supply increased stepwise by 0.3 kg/d till 8.5 kg/d on 28 DIM. Body weight, milk yield and feed intake were recorded daily. During lactation, cows were milked twice daily at ~0600 hours and ~1800 hours.

### Milk samples

Milk samples for analysis of fat, protein and lactose percentage (ISO 9622, Qlip, Zutphen, The Netherlands) were collected four times per week (Tuesday afternoon, Wednesday morning, Wednesday afternoon, and Thursday morning), Milk samples were analysed as a pooled sample per cow per week and used to calculate average fat, protein and lactose yield per week. Milk samples for metabolomics studies were collected at Friday morning each week. All samples were collected and stored at −20 °C until analysis. Milk production traits were averaged per week for week 2 and 7. Four milk samples were omitted because the dairy cows were suffering from mastitis in sampling week, three cows in week 2 and one cow in week 7.

### Energy intake and energy balance

Roughage and concentrate was supplied separately and daily intakes were recorded per individual cow using roughage intake control troughs (Insentec, Marknesse, the Netherlands). Energy balance was calculated per week according to the Dutch net energy evaluation (VEM) system, as the difference between net energy intake and the estimated net energy requirements for milk yield and maintenance^27^. Energy requirements for maintenance are based on body weight^27^. According to the VEM system, the daily requirement for maintenance is 42.4 VEM/kg^0.75^ of BW, the requirement for milk yield is 442 VEM/kg of FPCM. Energy intake and energy balance are expressed in kJ/kg^0.75^ per day, where kg^0.75^ indicates metabolic body size^27^.

### Mass Spectrum measurement and data processing

For quantification of metabolites, a targeted, standardized and quality controlled metabolic phenotyping was performed based on LC-QQQ-MS analysis. Milk serum was transferred to an Eppendorf tube, the fat layer was removed after centrifuge (12,000 rpm, 15 min, Eppendorf centrifuge 5424 with FA-45-24-11 fixed angle rotor). The milk samples were further prepared as in Lu *et al*.^34^. Measurements were performed with a triple quadrupole mass spectrometer (Shimadzu LC-QQQ-MS; LCMS-8040) using the PFPP method as described earlier^59,60^. The sample injection volume used was 1 μL, and a single analysis took 25 minutes.

### Statistical analyses

#### Multivariate analysis

The data obtained from LC-MS and milk production traits were first log transformed, then centered and scaled to unit variance. Principle component analysis (PCA) was performed on the processed data first for identifying outliers and observing general trends. Partial least squares discriminant analysis (PLS-DA) was applied to discriminate lactation weeks. Briefly, lactation week (week 2 and 7) was used as categorical variable Y, and milk yield, fat yield, protein yield, lactose yield, urea, fat- and protein-corrected milk (FPCM) as well as 52 milk metabolites were used as predictor variables X. Repeated double-cross validation was used to determine the optimal number of PLS components. Permutation test (5,000 permutations) was used asses the validity of PLS discriminant model and to avoid overfitting^61^. Partial least squares (PLS) regression was used to investigate the association between energy balance and milk metabolites and milk production traits. The energy balance of dairy cows was continuous variable Y, and milk yield, fat yield, protein yield, lactose yield, urea, fat- and protein-corrected milk (FPCM) as well as 52 milk metabolites in week 2 (or week 7) were used as observable variables X. Double cross-validation was used to determine the optimal number of PLS components. Permutation test (5,000 permutations) was used to assess the validity of PLS regression model and to avoid overfitting. Variable importance in projection (VIP) score were used to select the most contributing variable to the PLS model^62^.

#### Mixed models

To analyse the effect of energy balance, DPL and parity, top 15 variables with relatively higher VIP score in each dataset (week 2 or 7) were analysed using a Mixed Model approach, and DPL, parity, and their two-way interactions were included in the model as fixed effects. The statistical model used for milk metabolites and milk production traits was as follows:

$${{\rm{M}}}_{{\rm{jk}}}={\rm{\mu }}+{\rm{Energy}}\,{\rm{balance}}+{{\rm{DPL}}}_{{\rm{j}}}+{{\rm{Parity}}}_{{\rm{k}}}+{{\rm{Interactions}}}_{{\rm{jk}}}+{{\rm{\varepsilon }}}_{{\rm{jk}}}$$

Where M represents the observed level of milk metabolites and milk production traits. The mean is represented by μ. DPL_j_ represents the fixed class effect of DPL (j = 0 day, 30 days). Parity_k_ represents the fixed class effect of parity (k = 2nd parity, 3rd parity). Interactions_jk_ are presented as significant effect from Energy balance × DPL_j_ + Energy balance × Parity_k_ + DPL_j_ × Parity_k_, + Energy balance × DPL_j_ × Parity_k_. Non-significant interactions were excluded from the model via backward stepwise elimination if *P*-value was more than 0.10.

#### Stepwise regression

For obtaining reduced models with a maximum of 4 variables to estimate energy balance, top 14 variables from milk in week 2 (or week 7) with relatively higher VIP score in the PLS model were analysed using ten-fold cross-validation. Briefly, an *F*-test was constructed based on forward selection approach. *R*^2^ increases with the increased estimator in the model, however, the adjusted *R*^2^ increases only if the new term improves the model more than would be expected by chance. The model would be selected only with higher adjusted *R*^2^. The statistical model used to estimate energy balance is as follows:

$${\rm{Energy}}\,{\rm{balance}}={\rm{intercept}}+\sum _{j=1}^{N}{{\rm{M}}}_{j}+{\rm{error}}$$

where *N* is total number of milk metabolites and milk production traits (M_*j*_) used in to model to estimate energy balance, 1 ≤ *N* ≤ 6.

Multivariate analysis was performed with the R environment (version: 3.3.2), PLS-DA and PLS regression were performed with using the package “MixOmics”^63^, double cross-validation was executed with package “chemometrics”^64^. The correlation plots of data in week 2 (or week 7) were drawn with package “corrplot”. The Mixed Model was performed through PROC MIXED of SAS version 9.3 (SAS Institute, Inc., Cary, NC). The 10 fold cross-validation for obtaining reduced models was performed in R-project with package “DAAG”^65^.

## Electronic supplementary material

Supplementary information
