# Supplementary material for: Milk Metabolomics Data Reveal the Energy Balance of Individual Dairy Cows in Early Lactation
Source: Sci Rep. 2018 Oct 25;8:15828. doi: 10.1038/s41598-018-34190-4 (PMC6202381; doi:10.1038/s41598-018-34190-4)

Milk Metabolomics Data Reveal the Energy Balance of Individual Dairy Cows in Early Lactation

Wei Xu[1, 2], Jacques Vervoort[2*], Edoardo Saccenti[3], Renny van Hoeij[1], Bas Kemp[1] & Ariette van Knegsel[1*]

1. Adaptation Physiology Group, Department of Animal Sciences, 2. Laboratory of Biochemistry, 3. Laboratory of Systems and Synthetic Biology, Wageningen University & Research, Wageningen, the Netherlands

** Corresponding author. Email: jacques.vervoort@wur.nl; ariette.vanknegsel@wur.nl*

Principal Component Analyse

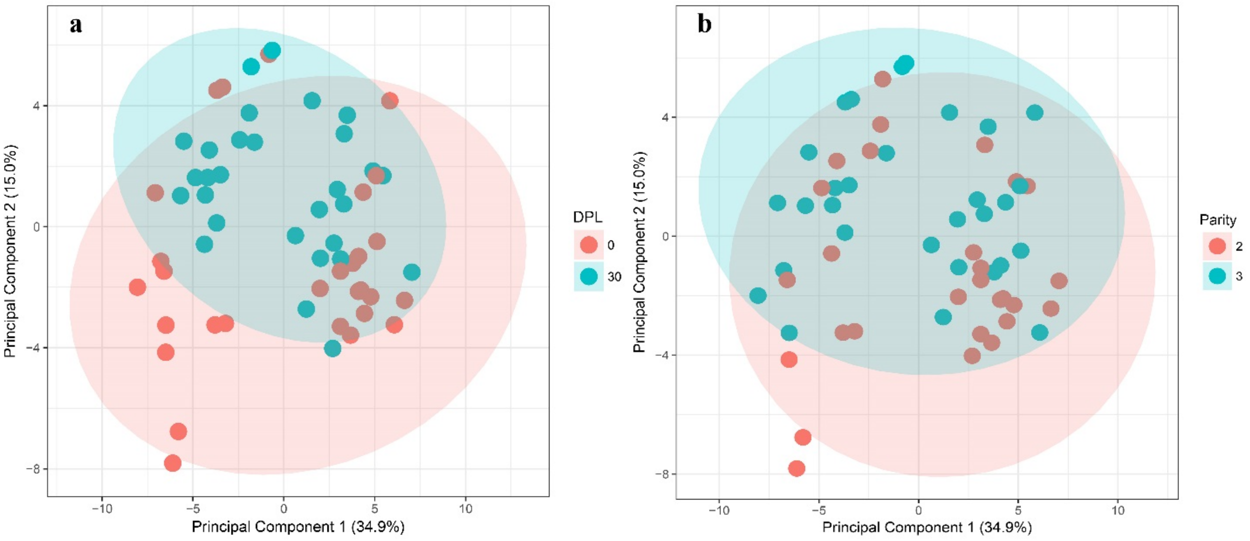

Supplementary Fig. S1. Principal component analyses to identify metabotypes, data were labelled with different dry period length (a) and parity (b).

Supplementary Fig. S2. Pearson correlations matrix among milk metabolites, milk production traits and energy balance of dairy cows in week 2 (a) and week 7 (b). Blank represents the *P*-value of correlation between two variables is insignificant (*P*-value > 0.05).

Abbreviation: cAMP, adenosine 3,5-cyclic monophosphate; AMP, adenosine monophosphate; CMP, cytidine monophosphate; FMN, Flavin mononucleotide; FPCM, fat- and protein-corrected milk production.

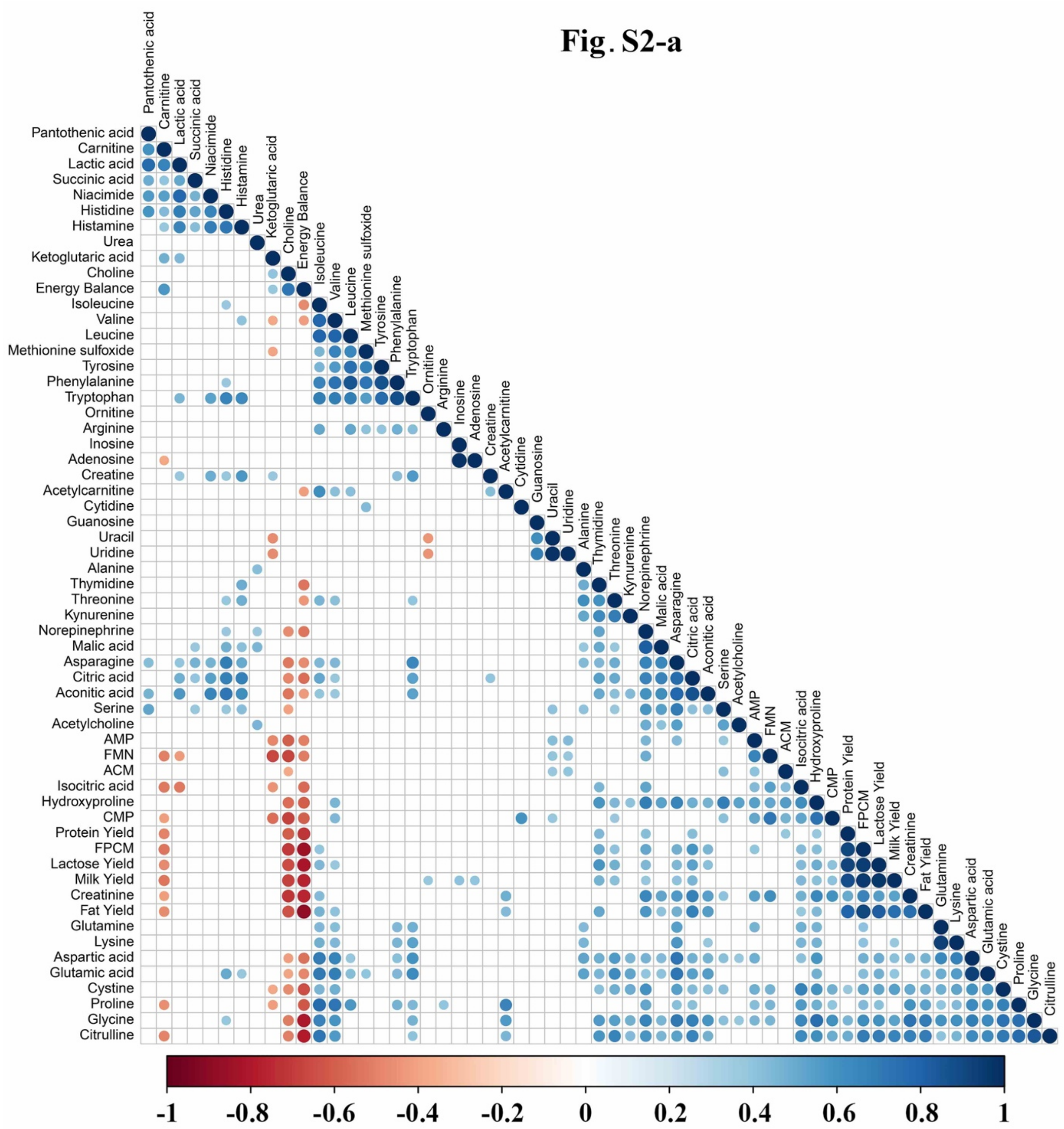
Fig. S2-a
Pantothenic acid
Carnitine
Lactic acid
Succinic acid
Niacimide
Histidine
Histamine
Urea
Ketoglutaric acid
Choline
Energy Balance
Isoleucine
Valine
Leucine
Methionine sulfoxide
Tyrosine
Phenylalanine
Tryptophan
Ornitine
Arginine
Inosine
Adenosine
Creatine
Acetylcarnitine
Cytidine
Guanosine
Uracil
Uridine
Alanine
Thymidine
Threonine
Kynurenine
Norepinephrine
Malic acid
Asparagine
Citric acid
Aconitic acid
Serine
Acetylcholine
AMP
FMN
ACM
Isocitric acid
Hydroxyproline
CMP
Protein Yield
FPCM
Lactose Yield
Milk Yield
Creatinine
Fat Yield
Glutamine
Lysine
Aspartic acid
Glutamic acid
Cystine
Proline
Glycine
Citrulline
-1
-0.8
-0.6
-0.4
-0.2
0
0.2
0.4
0.6
0.8
1

Fig.S2-b

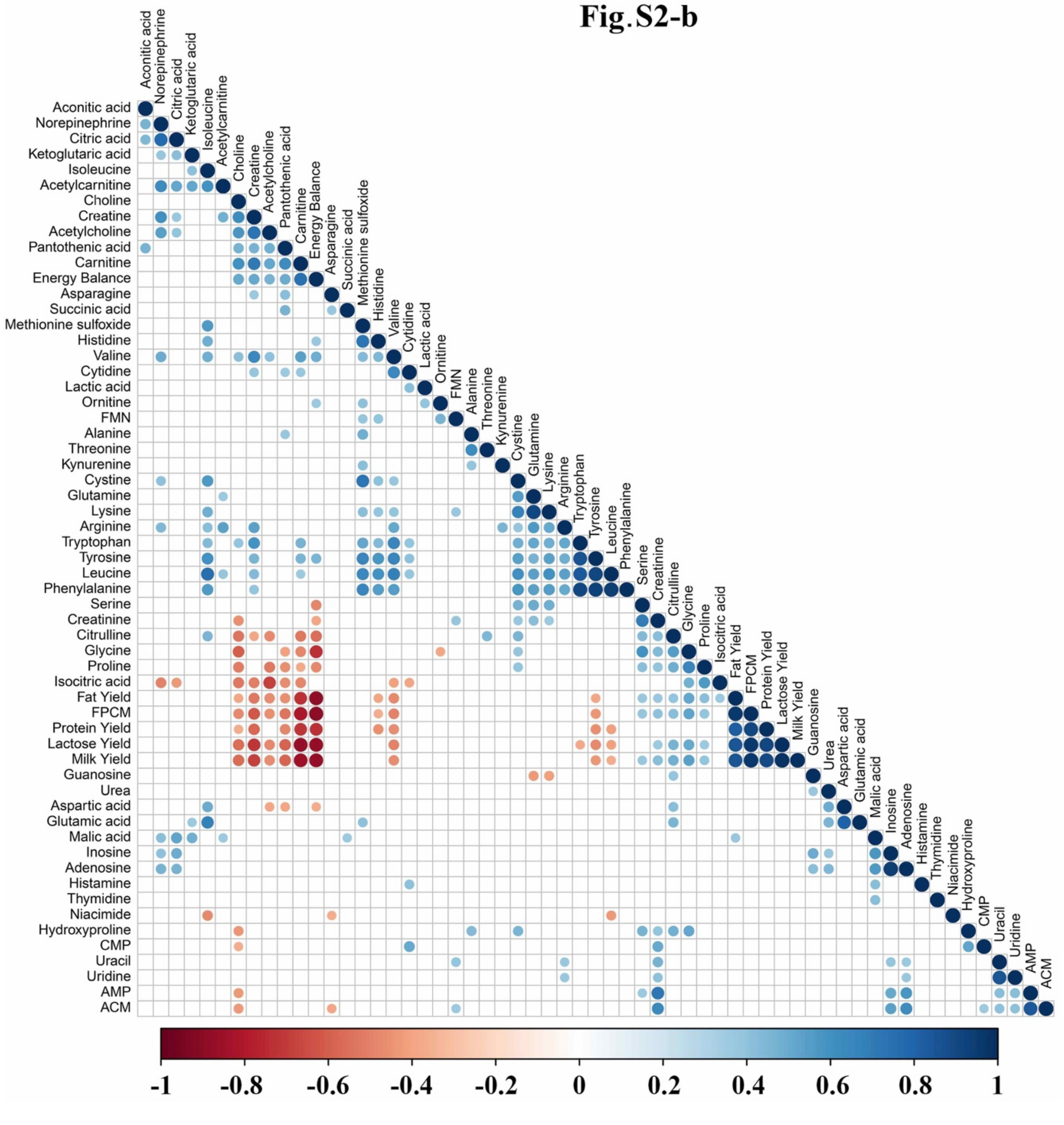
Aconitic acid
Norepinephrine
Citric acid
Ketoglutaric acid
Isoleucine
Acetylcarnitine
Choline
Creatine
Acetylcholine
Pantothenic acid
Carnitine
Energy Balance
Asparagine
Succinic acid
Methionine sulfoxide
Histidine
Valine
Cytidine
Lactic acid
Ornitine
FMN
Alanine
Threonine
Kynurenine
Cystine
Glutamine
Lysine
Arginine
Tryptophan
Tyrosine
Leucine
Phenylalanine
Serine
Creatinine
Citrulline
Glycine
Proline
Isocitric acid
Fat Yield
FPCM
Protein Yield
Lactose Yield
Milk Yield
Guanosine
Urea
Aspartic acid
Glutamic acid
Malic acid
Inosine
Adenosine
Histamine
Thymidine
Niacimide
Hydroxyproline
CMP
Uracil
Uridine
AMP
ACM
-1
-0.8
-0.6
-0.4
-0.2
0
0.2
0.4
0.6
0.8
1

Supplementary Fig. S3. The effect of significant two-way interaction between energy balance (EB) and different treatments, including dry period (DPL) and parity on in week 2 glycine (A-1, A-2, A-3), Creatinine (B), Choline (C), adenosine 3,5-cyclic monophosphate (cAMP) (D), Hydroxyproline (E), citric acid (F), milk yield (G), lactose (H), fat yield (I-1, I-2), protein (J-1, J-2), FPCM (K-1, K-2).

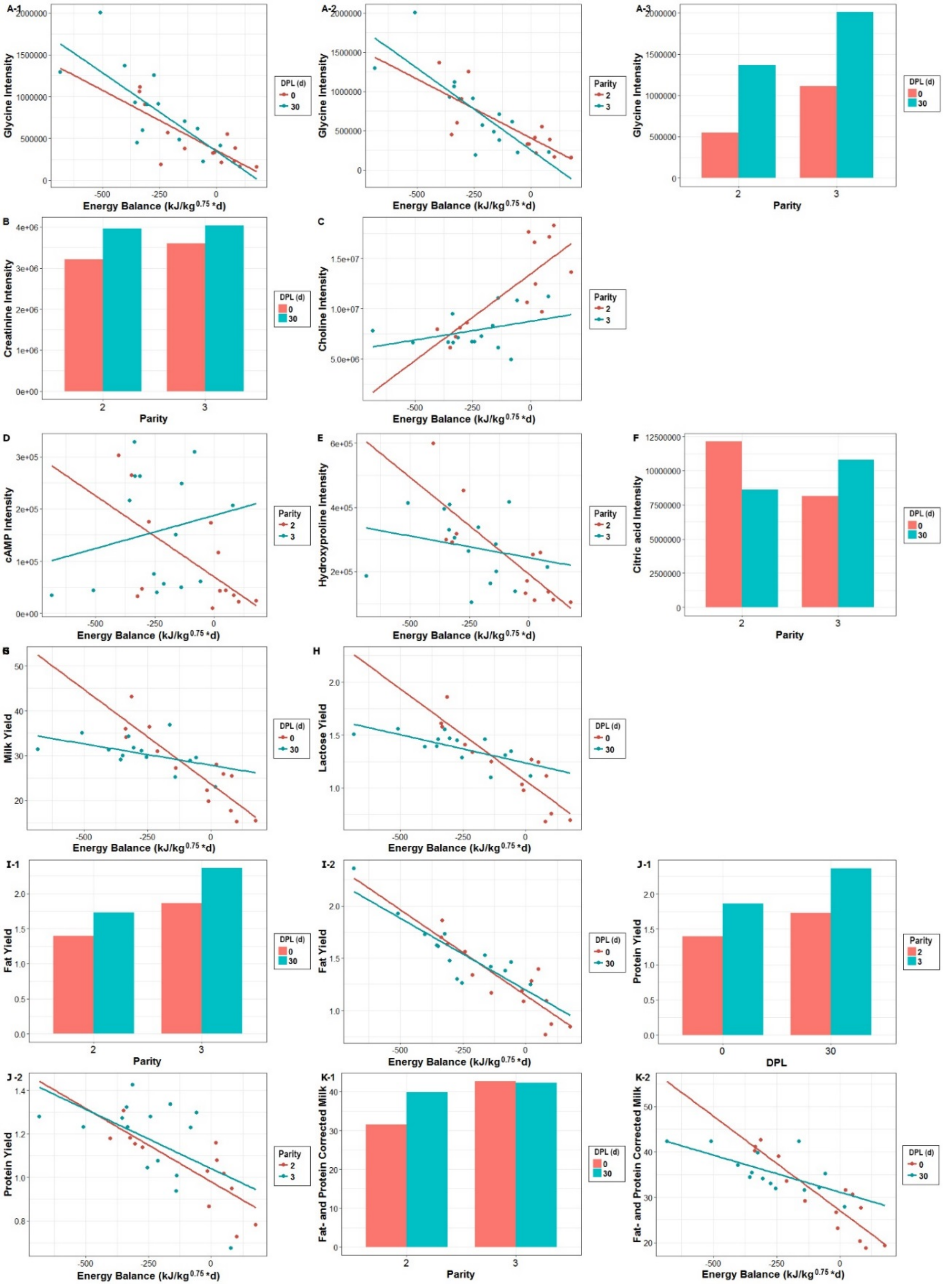

Supplementary Fig. S4. The effect of significant two-way interaction between EB and different treatments, including dry period (DP) and parity in week 7 on glycine (A-1, A-2), Citrulline (B-1, B-2), Tyrosine (C-1, C-2, C-3), lactose (D), protein (E), FPCM (F).

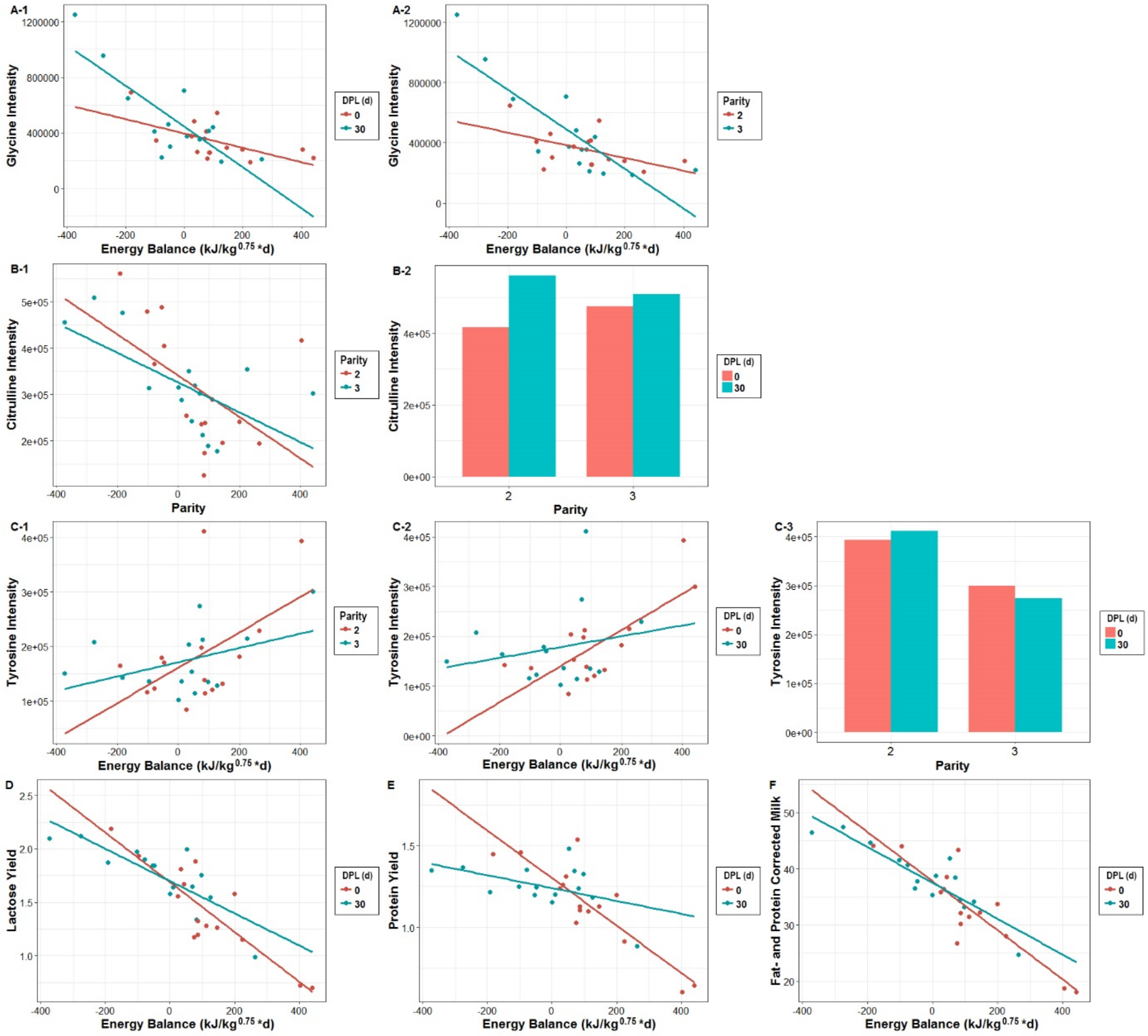

Supplement: Supplementary file 1 — Supplementary information [file 41598_2018_34190_MOESM1_ESM.pdf]
